# Skin region images extracted from 3D total body photographs for lesion detection

**DOI:** 10.1038/s41597-025-05483-x

**Published:** 2025-08-18

**Authors:** Anup Saha, Joseph Adeola, Nuria Ferrera, Adam Mothershaw, Gisele Rezze, Séraphin Gaborit, Brian D’Alessandro, Robert Voskanyan, Gyula Szabó, Balázs Pataki, Hayat Rajani, Sana Nazari, Hassan Hayat, Laura Serra-García, Clare Primiero, Serena Bonin, Iris Zalaudek, H. Peter Soyer, Josep Malvehy, Rafael Garcia

**Affiliations:** 1 Computer Vision and Robotics Research Institute, University of Girona, Girona, Spain; 2 Dermatology Department, Hospital Clínic Barcelona, Universitat de Barcelona, Barcelona, Spain; 3 Institut d’Investigacins Biomèdiques August Pi i Sunyer (IDIBAPS), Barcelona, Spain; 4 Frazer Institute, The University of Queensland, Dermatology Research Center, Brisbane, Australia; 5 Department of Medical Sciences, University of Trieste, Trieste, Italy; 6 ISAHIT, Paris, France; 7 Canfield Scientific, Inc., Parsippany, New Jersey USA; 8 V7, London, United Kingdom; 9 HUN-REN Institute for Computer Science and Control, Budapest, Hungary; 10 Department of Dermatology and Venereology, University of Trieste, Trieste, Italy; 11 Dermatology Clinic, ASUGI, Maggiore Hospital, Trieste, Italy; 12 Dermatology Department, Princess Alexandra Hospital, Brisbane, QLD Australia

**Keywords:** Melanoma, Whole body imaging, Basal cell carcinoma, Squamous cell carcinoma

## Abstract

Artificial intelligence has significantly advanced skin cancer diagnosis by enabling rapid and accurate detection of malignant lesions. In this domain, most publicly available image datasets consist of single, isolated skin lesions positioned at the centre of the image. While these lesion-centric datasets have been fundamental for developing diagnostic algorithms, they lack the context of the surrounding skin, which is critical for improving lesion detection. The iToBoS dataset was created to address this challenge. It includes 16,954 images of skin regions from 100 participants, captured using 3D total body photography. Each image roughly corresponds to a 7 × 9 cm section of skin with all suspicious lesions annotated using bounding boxes. Additionally, the dataset provides metadata such as anatomical location, age group, and sun damage score for each image. This dataset was designed with the aim of facilitating the training and benchmarking of algorithms, in order to enable early detection of skin cancer and deployment of this technology in non-clinical environments.

## Background & Summary

Skin cancer is the most prevalent form of cancer globally, with Melanoma (MEL), Basal Cell Carcinoma (BCC), and Squamous Cell Carcinoma (SCC) representing the most common malignancies. These cancers have become increasingly prevalent, underscoring the critical need for effective diagnostic and monitoring solutions^1^. Traditionally, skin cancer diagnosis has relied heavily on the dermatologist’s expertise and the use of dermatoscopy. Dermatoscopy is a non-invasive approach that uses a dermoscope to enhance the view of the sub-macroscopic structures in pigmented skin lesions, which vary widely across dermatological conditions^2,3^. As a result, they provide critical cues, not only for the visual examination of suspicious lesions by trained dermatologists but also for AI-based diagnostic tools^4–11^ that aid in differentiating between malignant and benign skin conditions. Consequently, most publicly available skin lesion datasets for training AI models are dominated by dermoscopic images^12–15^.

However, while dermoscopy has resulted in improved diagnostic precision, its reliance on specialised equipment and skilled practitioners poses significant barriers to early detection and triage. In such contexts, computer-aided diagnosis systems that utilise conventional camera images offer a more practical and scalable solution. These systems, designed to work with standard smartphone cameras, can be deployed in resource-constrained environments, enabling non-specialist healthcare providers - such as general practitioners or community health workers - to identify potentially malignant lesions. 3D total-body photography (3D-TBP) is increasingly gaining recognition as a foundational tool in this regard^16–18^. Such imaging systems use a coordinated setup of cameras to capture comprehensive, high-resolution views of almost the entire skin surface in a single session, providing a detailed and holistic representation of a patient’s skin. This not only provides a broader perspective for lesion analysis by incorporating the surrounding skin area that might be otherwise excluded when assessing a single localised lesion, but it can also facilitate longitudinal tracking of lesions over time to monitor disease progression and treatment response.

Canfield’s VECTRA WB360 is a state-of-the-art 3D-TBP system that provides automatic lesion detection capabilities. Recently, the International Skin Imaging Collaboration (ISIC) used this system to compile a lesion classification dataset^19^ consisting of lesion-centric crops extracted from 3D-TBP scans as part of their lesion classification challenge, ISIC 2024, which garnered a huge response. However, the system’s automated lesion detection occasionally introduced false positives such as knuckles, nipples, bellybuttons, and tattoos, leading to all images being manually reviewed to remove erroneously classified samples and refine the dataset^19^. In addition, the system was also observed to over-identify solar damage lesions. Given the above, it can be asserted that there remains a lack of publicly available datasets for training AI models to improve lesion detection. We therefore present a novel dataset comprising 16,954 high-resolution images taken from two different sites: Hospital Clinic Barcelona, Spain and The University of Queensland, Brisbane, Australia. In terms of contribution, the Barcelona site comprised 51 patients and the Brisbane site 49. These images are tiles of the patients’ skin surface captured using the VECTRA WB360 3D-TBP system (Canfield Scientific) and extracted directly from the original 2D camera images, with the 3D avatar serving as a coordinate system for precise region identification. The dataset comprises images from diverse anatomical locations, including the torso, arms and legs, but excluding the face to preserve patient anonymisation. Each image is annotated with bounding boxes that enclose skin lesions, providing a rich dataset for training AI models for lesion detection. By capturing a diverse set of lesions across different anatomical regions and patient demographics, the dataset reflects the variability seen in real-world clinical practice. The images are also accompanied by metadata such as the patient’s age, anatomical region, and sun damage score corresponding to each image. These parameters were carefully chosen to provide additional context for AI models, helping them distinguish between lesions and healthy skin by more effectively accounting for demographic and environmental factors that influence these conditions.

While the images in this dataset were captured in clinical settings with standardised lighting and positioning, they provide valuable resources for extending lesion detection to non-clinical environments such as smartphone-based applications. The high-quality annotations and consistent imaging make these images ideal for pretraining lesion-localisation networks, which can then be fine-tuned on smaller datasets of smartphone photos^20^.

This dataset represents a significant advancement compared to existing publicly available skin lesion datasets, as illustrated in Table 1. While datasets like HAM10000^13^, BCN20000^14^, and the ISIC SLICE-3D^19^ provide valuable resources for classification tasks, they typically feature single, isolated lesions with image-level diagnostic labels. In contrast, our dataset provides contextual skin region tiles that frequently contain multiple lesions in their natural anatomical setting, with precise bounding box annotations for each lesion. This approach addresses the critical need for datasets specifically designed for lesion detection rather than classification, enabling the development of algorithms that can identify and localise lesions within broader skin regions—an essential capability for comprehensive skin examination systems.

**Table 1:** Comparative overview of some publicly available datasets and the iToBoS dataset, highlighting annotation types and key machine learning tasks.

Dataset	Images	Annotation type	Multiple lesions per image?	Primary ML task	Image type
iToBoS (ours)	16,954 (tiles)	Bounding boxes for every lesion	Yes	Detection / localisation	Clinical (skin region images from 3D avatars)
HAM10000 (2018)^13^	10,015	Image-level diagnosis label	No	Classification	Dermoscopic, lesion-centric
BCN20000 (2024)^14^	19,424	Image-level diagnosis label	No	Classification	Dermoscopic, lesion-centric
PAD-UFES-20 (2020)^25^	2,698	Image-level diagnosis label	No	Classification	Clinical (smartphone), lesion-centric
ISIC SLICE-3D (2024)^19^	400,000 (crops)	Image-level diagnosis label	Typically 1	Classification	Clinical (lesion crops from 3D avatars)

## Methods

### Acquiring ethical authorisation for data sharing

This study received ethical approval from two institutions: the Human Research Ethics Committee at The University of Queensland, Brisbane, Australia (approval ID: 2022/HE001866) and the Hospital Clinic Barcelona Research Ethics Committee, Spain (approval ID: HCB/2022/1051). The research was registered with ClinicalTrials.gov (ref.NCT05955443) and conducted in accordance with Good Clinical Practice guidelines. All study procedures adhered to the ethical principles outlined in the Declaration of Helsinki (1964).

### Participant’s consent acquisition

All participants provided written, specific consent for both participation and data sharing. In Brisbane, between October 2022 and April 2023, individuals who had undergone 3D-TBP imaging during previous research visits (September 2016 to February 2020) were contacted via email to provide online consent for the use of their existing images. In Barcelona, participants consented to new imaging as part of their clinical evaluation. This consent allowed de-identified images with minimal demographic data to be included in a public research dataset. No waiver of consent was required as all participants actively consented.

### Dataset Generation

**Fig. 1:**
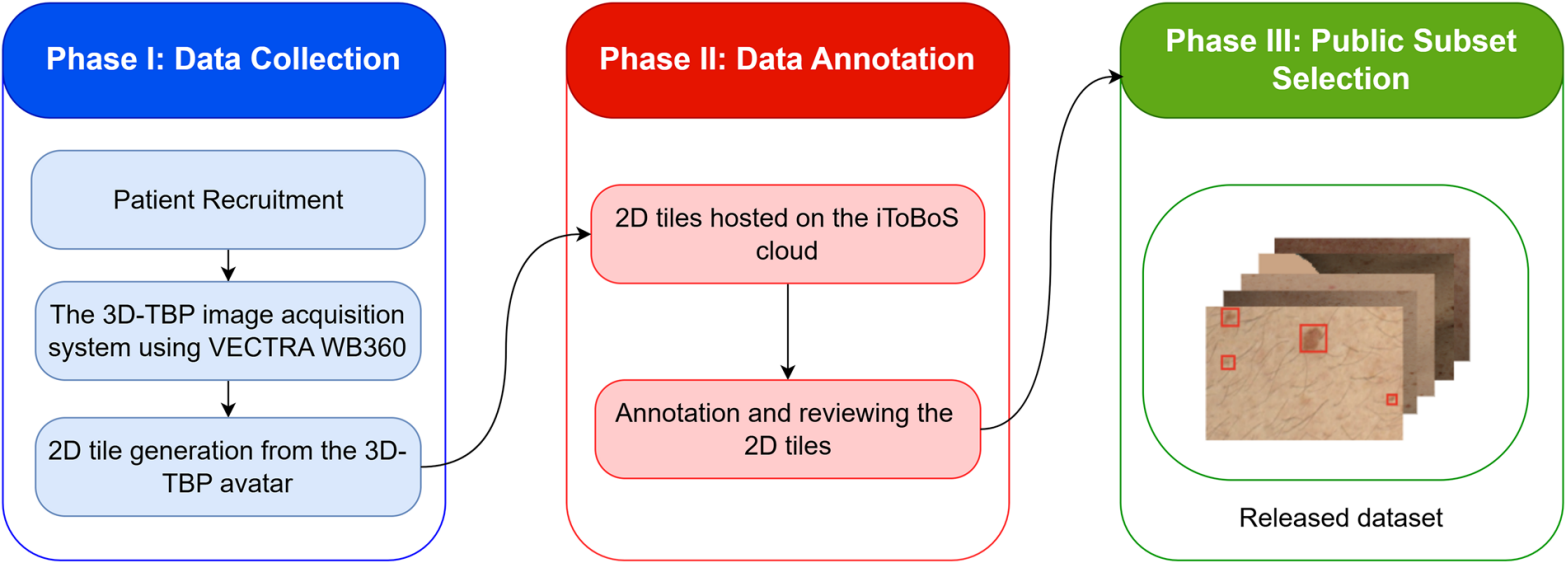
Three-phase methodology for dataset generation: (**i**) data collection through 3D-TBP imaging and 2D tile extraction, (**ii**) data annotation and expert review, and (**iii**) careful selection of a representative public subset released.

Following ethical approval and consent acquisition, the dataset generation process involved three key phases: (i) data collection, (ii) data annotation, and (iii) public subset selection, as summarised in Fig. 1. Phase (i), data collection, began with patient recruitment at two clinical sites, followed by capturing 3D-TBP images using the VECTRA WB360 scanner and the extraction of 2D tiles using 3D avatar mapping. Phase (ii), data annotation, comprised hosting these tiles on the iToBoS cloud platform, annotation, and quality control. Finally, phase (iii) involved the careful selection of a public subset for release. Further details of each phase are provided in the following sections.

### Phase I: Data collection

#### Patient recruitment

The study utilised different data collection approaches in two locations. In Brisbane, Australia, we leveraged existing 3D-TBP images from individuals who had been imaged using the VECTRA WB360 system during previous research visits between September 2016 and February 2020.

In Barcelona, Spain, active recruitment occurred from January 2023 to March 2024. Individuals with personal or family history of melanoma, or atypical nevus syndrome, were invited for clinical evaluation at Hospital Clinic Barcelona. These participants underwent new 3D-TBP imaging using the VECTRA WB360 system specifically for this study.

To ensure comprehensive data collection and tracking, each participant was assigned a unique identifier, with some participants undergoing multiple imaging sessions over time. Additionally, all participants completed detailed questionnaires capturing demographic, sun protective behaviour and phenotypic information, along with their relevant medical history.

#### The 3D-TBP image acquisition system using VECTRA WB360

Each participant underwent total body imaging, which was performed using the VECTRA WB360 system. This imaging system was designed for comprehensive skin documentation through efficient, high-resolution capture. The system utilises 92 fixed cameras (arranged in 46 stereo pairs) and xenon flashes to photograph the entire exposed body in a single capture. To ensure standardised image quality, participants were instructed to maintain a specific anatomical position while the system captured detailed images using both polarised and non-polarised lighting.

The captured images were processed by the VECTRA software to create a precise 3D avatar, enabling full 360-degree rotation for thorough examination of all body surfaces, including curved areas, which are often challenging to assess with traditional 2D imaging^21^.

All imaging data was saved in DX2 format, a well-known file type used for various applications, including medical imaging and dermatological assessments. Each DX2 file contains the original 2D images, the reconstructed 3D avatar, and all associated metadata required for dermatological assessment.

#### 2D tile generation from the 3D-TBP avatar

The process of extracting images corresponding to 2D tiles of the skin surface was accomplished using *WbTilingTool*, a specialised module within the *VectraDBTool* developed by Canfield Scientific. While the VECTRA system initially captures multiple 2D images to construct a 3D avatar (as described in the previous subsection), the tile extraction process works with the relationship between these components: it uses the 3D avatar as a spatial reference map to identify regions within the original 2D camera images. This approach maintains the native image quality of the original photographs by working directly with the raw camera images rather than converting the 3D avatar back to 2D, which could introduce projection artefacts. To preserve this authenticity, no background modification was performed; similarly, no colour correction or contrast enhancement was applied to the images.

**Fig. 2:**
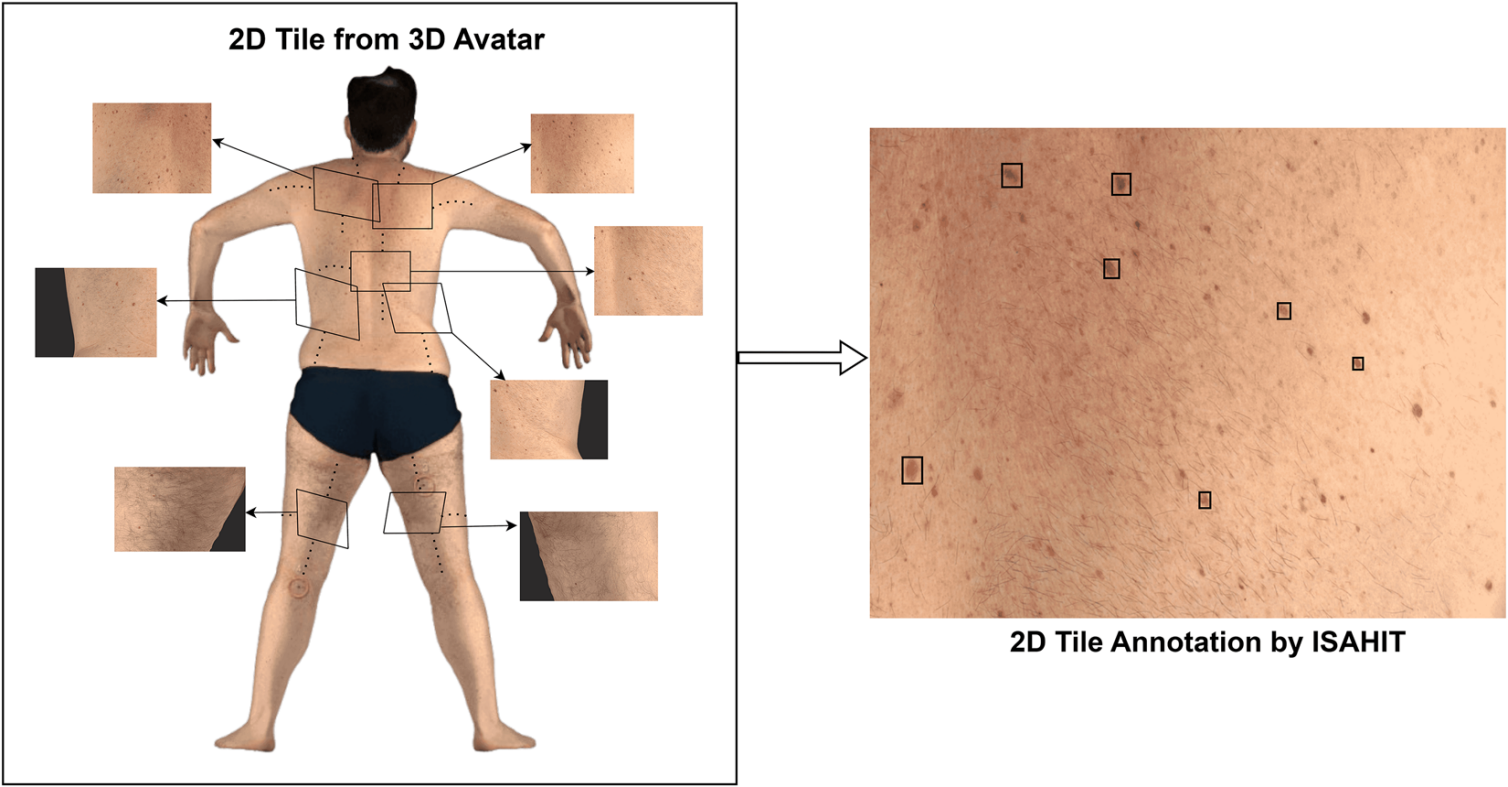
2D tiles generation from 3D-TBP: The iToBoS dataset consists of the patients’ skin surface tiles extracted from original 2D camera images using 3D avatar mapping for region identification. Subsequently, these images were annotated by ISAHIT and quality checked by clinicians on the V7 Darwin platform.

The process begins with an algorithm that analyses the front-view outline of the 3D avatar to identify different anatomical regions. This is done by creating a flat, front-facing silhouette of the body and then using software to divide this silhouette into distinct anatomical regions. Once these regions are identified on the silhouette, they are mapped back to the corresponding areas in the original camera images. For optimal imaging with the VECTRA system, participants were positioned in a specific pose with arms open and legs spread out, as shown in Fig. 2. This specific pose is necessary for the VECTRA scanner to capture the entire skin surface. In cases where this required pose was not maintained (for example, if the arms were placed by the side), the algorithm might fail to correctly classify certain body parts. These unidentifiable regions are labeled with an “Unknown” class.

**Fig. 3:**
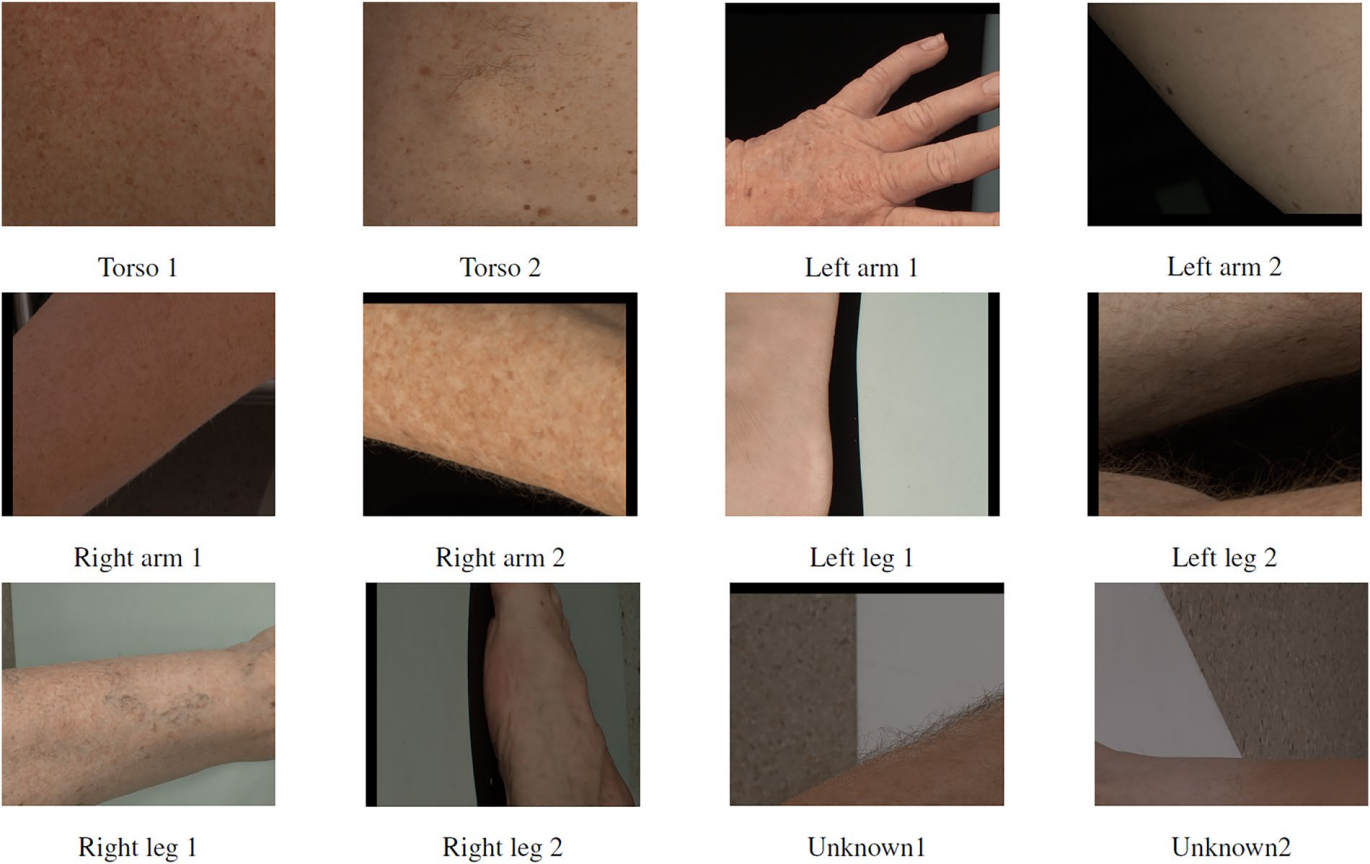
Sample 2D tiles illustrating the six anatomic region categories: torso (anterior and posterior), left arm, right arm, left leg, right leg, and unknown regions.

For each skin region, the corresponding 2D camera image captured from the angle most perpendicular to the skin surface is then divided into a 7 × 6 grid of equal-sized tiles. Each tile overlaps with its adjacent tiles by 45 pixels on each side. This deliberate overlap ensures that skin lesions located near the edges of tiles aren’t partially cut off or divided between tiles. Without this overlap, lesions positioned at tile boundaries might be incompletely captured in any single tile, making accurate detection and analysis difficult. Due to some differences in the cameras used in the VECTRA systems across clinical sites, the raw camera image dimensions vary slightly, resulting in four main tile dimension variants across the dataset: 1090 × 890, 1090 × 894, 1094 × 894, and 945 × 771 pixels. During the tile extraction process from the raw camera images, each tile inherits the anatomical class of its source region and falls into one of six categories as summarised in Table 2: torso, left arm, right arm, left leg, right leg, or unknown. Figure 2 illustrates an example of 2D tiles in relation to where they originate in the 3D avatar, while representative examples of image tiles from each category of anatomical regions are presented in Fig. 3.

**Table 2:** Anatomical Region Categorisation for Image Classification.

Region	Description
Torso	Anterior and posterior regions of the trunk, excluding limbs
Left arm	Hand, forearm, and upper arm up to the shoulder
Right arm	Hand, forearm, and upper arm up to the shoulder
Left leg	Foot, lower leg (shin and calf), and upper leg (thigh)
Right leg	Foot, lower leg (shin and calf), and upper leg (thigh)
Unknown	Cases where the system cannot automatically identify the anatomic region, typically occurring when patient poses are non-standard (e.g., arms at sides, legs not spread) or when technical limitations prevent accurate region labeling.

In addition to tile extraction, *WbTilingTool* was designed to streamline image processing by automatically detecting the patient’s head in 3D avatars. This detection allows the system to identify facial regions in the corresponding camera images and inpaint them before tile extraction. When tiles are subsequently extracted from these pre-processed images, they automatically exclude facial features, ensuring patient anonymity without altering other aspects of the skin appearance. Its robust functionality includes batch processing, which enables the simultaneous handling of multiple scans from various patients, significantly improving efficiency for large datasets.

The anatomical categorisation, along with extracted metadata such as age and sex-at-birth from participant questionnaires, was crucial for ensuring a balanced distribution when selecting the subset for public release, as detailed later. Also, the 3D spatial coordinates associated with each tile were also removed to enhance privacy. This critical step ensured that the tiles could not be used to reconstruct a 3D avatar of the patient, eliminating any potential risk of re-identification through spatial mapping. Additionally, the file names of the extracted 2D tiles were randomised, further reducing the risk of traceability or association with the original scans. To ensure complete anonymisation, scars, tattoos, and jewellry remnants in the tiles were masked out during the annotation stage. These steps ensured that patient identities were thoroughly protected, compliant with GDPR guidelines for protecting patient privacy.

### Phase II: Data annotation

The extracted 2D tiles were annotated by a team of 25 annotators coordinated by Isahit, a company specialising in annotation services across various domains, including medical and image-based data. The team, which comprised medical students, nurses, and doctors, was divided into two distinct cohorts with separate responsibilities: labelers and reviewers. Labelers focused exclusively on the initial annotation of images, while reviewers were dedicated solely to quality assurance of those annotations. The reviewer cohort was specifically selected for their superior quality assessment capabilities and more extensive experience with this particular annotation project. All annotators received specialised training to accurately identify and label skin lesions, and this two-tier structure ensured independent verification of all annotations before acceptance.

The *labellers* began by assessing the level of sun damage visible on each tile, assigning a severity tag from 1 to 3, with level 1 indicating low and level 3 indicating high sun damage. Each lesion was then outlined using a bounding box and its colour was annotated to enhance characterisation. Each tile was also tagged if it contained any tattoos or identifiable marks, thereby providing an additional layer of verification for challenging cases and enabling their subsequent annotation and masking. Finally, the *reviewers* checked each tile manually to verify the accuracy of the annotations. If a tile met the quality standards, it was marked as complete; otherwise, it was sent back to the *labellers* for re-annotation.

#### 2D tiles hosted on the iToBoS cloud

The iToBoS cloud, managed by the Computing and Automation Research Institute, HUN-REN SZTAKI, played a crucial role in the annotation process by taking on two key responsibilities: providing secure storage for the 2D tiles, and managing the transfer of these data to and from the V7 Darwin annotation platform. This process was handled via a NextCloud service, supported by HUN-REN’s cloud infrastructure. NextCloud allowed for seamless data uploads and management within a structured directory system, with strict authentication and authorisation protocols to ensure that only authorised personnel could access these files.

Once the files to be annotated had been allocated to specific folders on the NextCloud platform, the upload scripts were used to create datasets on the V7 Darwin platform. Then, the Isahit annotators used these datasets to perform the necessary annotations on the tiles. Following this, dermatologists verified the Isahit annotation on the V7 Darwin platform. After all datasets had been annotated and verified, the annotations were downloaded from the V7 Darwin platform back into designated folders on NextCloud.

This workflow provided a flexible and asynchronous approach, facilitating the smooth transfer of primary data to NextCloud, as well as the generation and retrieval of derivative data, including the annotations, back into the system.

#### Annotation and reviewing the 2D tiles

The V7 Darwin platform was used to annotate the tiles. This platform provided facilities for viewing the tiles and included a pre-defined set of tools for both polygonal annotation of lesions and assigning a sun damage score to each tile. Assessing sun damage provided additional information for lesion analysis, as areas with higher sun damage typically presented a greater risk for developing skin lesions and influenced their appearance and characteristics. A circular “ruler” with a diameter of 2.5 mm was also provided with each tile to help the annotators determine the size of the lesions being annotated. In this dataset, a minimum threshold of 2.5 mm was established because, although smaller lesions may occasionally be melanomas, they are less common and often present significant diagnostic challenges^22^. In addition, the V7 Darwin platform enabled the design and deployment of custom workflows. These workflows allowed for the assignment of lesion annotation and review tasks to Isahit’s *labellers* and *reviewers*, followed by a final review by clinicians. The platform also facilitated simultaneous operations by all participants in the workflow, allowing them to annotate lesions, add tags, accept or reject annotations, and provide feedback, thereby streamlining the annotation process and ensuring efficiency across tasks. The workflows were modular and were adjusted throughout the project to achieve the highest possible level of accuracy. An example of this was that requirements for multiple reviewers in sequence could be introduced as needed.

**Fig. 4:**
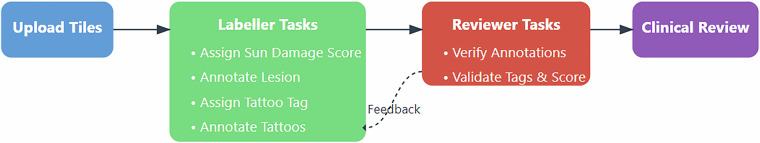
V7 Darwin platform annotation workflow illustrating the sequential process from tile upload to clinical review. Labellers perform sun damage scoring, tattoo tagging, and lesion annotation, while reviewers verify the completeness and accuracy of all labeller tasks. The dashed line represents the feedback loop for refinements.

**Fig. 5:**
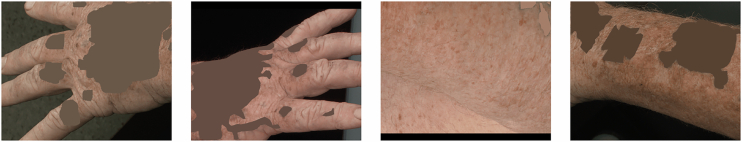
Examples of 2D tiles where tattoos were inpainted during the annotation phase to preserve patient privacy while maintaining skin features for lesion analysis.

The annotation workflow began with the tiles being uploaded to the V7 Darwin platform, as shown in Fig. 4. Within the designed workflow system, *labellers* were assigned batches of images to process. The annotation process differed by feature type: lesions were annotated manually by drawing bounding boxes, while tattoos, scars and jewellries were processed using V7’s “Auto Annotate” feature. This feature employed a general-purpose pre-trained segmentation model to automatically generate initial polygons, which were subsequently refined by labellers to ensure accurate delineation of the tattoos, scars and jewellries. The segmentation model, developed by V7, was trained on a diverse dataset of over 3 million items spanning various image types and medical data, enabling robust instance segmentation polygons across different visual contexts. The generated polygons were then used to mask these areas in the tiles. The sun damage assessment was performed at the image level, with labellers assigning appropriate tags based on visual inspection. Once the annotation process was completed for a batch, it was automatically routed to the next available *reviewer* who would conduct a quality control check for annotation accuracy. Across all processed batches, approximately 12% of annotations required modification during this review stage. Following this, three dermatologists independently reviewed the revised annotations, resulting in an additional 9% of annotations being further modified based on their expert feedback. An example of masked (inpainted) tattoos is provided in Fig. 5, which depicts certain examples of tiles containing masked tattoos.

### Phase III: Public Dataset Selection

To select an optimal data subset for public release, we began by analysing potential dataset biases. One extreme category was identified: participants under 30 years of age, which comprised just two patients. These outlier cases were initially set aside to be strategically distributed throughout the final training and test sets, ensuring that rare but clinically significant cases were preserved in the public release. For the remaining tiles, we adapted the *Wallace rule of nines*^23^, a method traditionally used for assessing burn surface area in dermatology, to establish sampling proportions across anatomical regions. This approach allocated 37.5% of the samples to the torso, 17% to each arm, 13.5% to each leg, and 0.5% to unknown cases, balancing practical considerations with anatomical representation while maintaining clinical relevance.

**Fig. 6:**
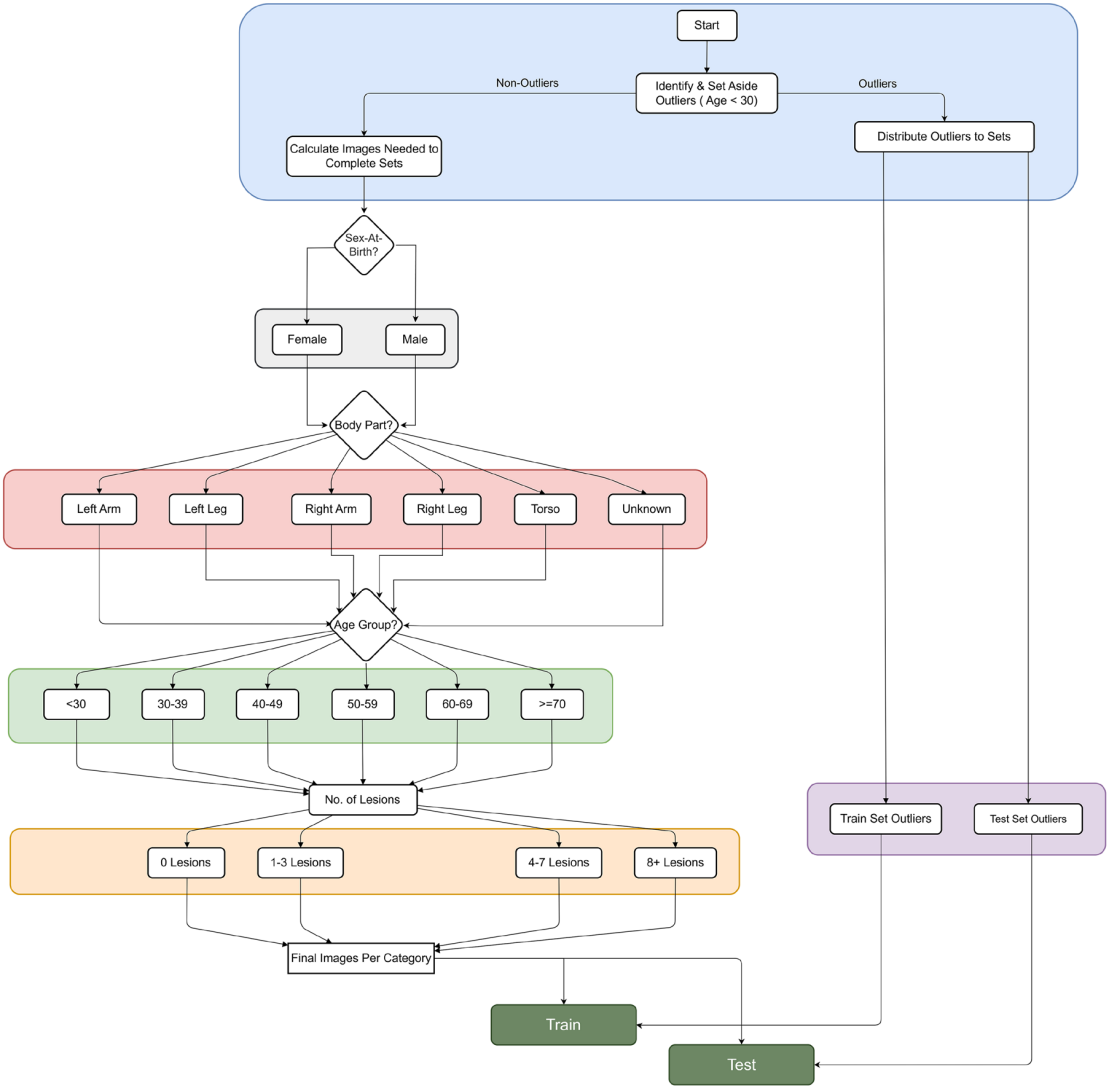
Hierarchical stratified sampling workflow for balanced subset selection. The process begins by setting aside outlier cases (age < 30), then splits the remaining data by sex-at-birth, anatomical region, presence of lesions, and finally applies random sampling within each stratum. The outlier cases are strategically allocated to the training and test sets to ensure representation of rare but clinically significant cases.

The selection process followed a hierarchical stratified sampling approach, as shown in Fig. 6, to ensure balanced representation across multiple dimensions. This systematic workflow guided the sampling through successive stages of categorisation, starting with sex-at-birth divisions and proceeding through anatomical regions and lesion presence. Within each resulting stratum, random sampling was performed to meet target counts while maintaining a proportional representation. The previously identified outliers were then integrated into the final dataset, ensuring that both common and rare presentations were available for model development and evaluation.

## Data Records

### Data accessibility

The dataset is available on Figshare^24^ and is organised in a manner that facilitates easy navigation and usability for research purposes. The dataset is released under a Creative Commons Attribution (CC-BY 4.0) license, in accordance with the licencing terms and conditions agreed upon by the providing institutions. It comprises categorised image tiles in PNG format and their corresponding annotations in two formats: text files using the YOLO format and JSON files using the COCO format. Since this is a single-label dataset, all annotations are assigned the label ‘0’.

The dataset is provided as a single unified collection, with a metadata file in CSV format that provides comprehensive information for each image, including the train/test split designation, anatomical location, patient demographics (age and sex at birth), sun damage score, and an inpainted boolean flag (TRUE/FALSE) indicating whether the image contains inpainted regions for privacy protection.

**Fig. 7:**
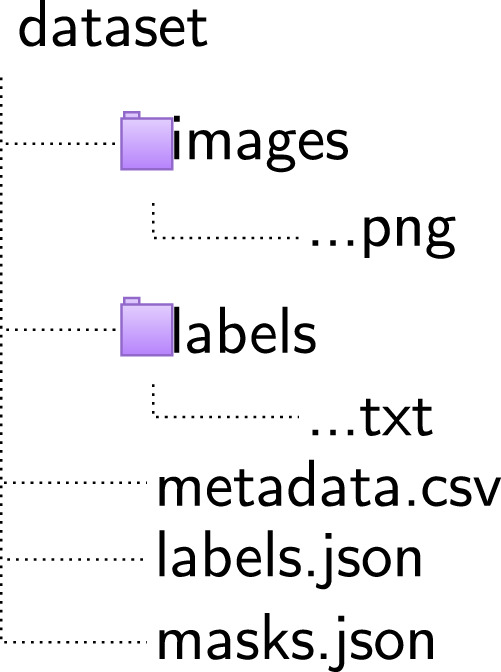
Dataset directory structure illustrating the unified organisation of image tiles, annotations (YOLO and COCO formats), metadata file, and mask information for the iToBoS dataset.

Additionally, a unified mask file is provided for all images with inpainting. The mask JSON file contains an *info* section and two arrays. The first array, *images*, lists only those PNG files whose entry in the metadata sheet is marked *inpainted = TRUE*; each image entry stores a unique integer *id* and the *file_name*. The second array, *annotations*, holds one record for every inpainted polygon: it stores a unique *id*, the parent *image_id*, and a single *mask* list (this field replaces the usual COCO *segmentation*). The *mask* list is a flattened sequence of vertex coordinates [*x*_1_, *y*_1_, *x*_2_, *y*_2_, …] that outlines an inpainted area—face, tattoo, scar, or jewellery remnant. No width, height, category or bounding-box fields are included, so the file simply maps each polygon to its image while remaining compact and easy to parse. Researchers can therefore locate, crop, or ignore the masked regions according to the needs of their own pipelines. The complete organisation of the dataset is illustrated in Fig. 7.

### Data description

The dataset consists of 8,473 images in the training set and 8,481 images in the test set. In the training set, 6,723 images contain lesions (2,412 from Barcelona and 4,311 from Brisbane) and 1,750 images are without lesions (770 from Barcelona and 980 from Brisbane). Similarly, the test set contains 6,750 images with lesions (3,633 from Barcelona and 3,117 from Brisbane) and 1,731 images without lesions (914 from Barcelona and 817 from Brisbane). To better illustrate the composition of the dataset, we present a comprehensive breakdown of the dataset characteristics is presented in Table 3.

**Table 3:** Overall dataset characteristics: distribution of lesion presence, anatomical locations, patient demographics (n = 49 patients in training, n = 51 patients in test), and annotation counts across training and test sets by data collection sites.

Category	Training set			Test set		
	Barcelona (count(%))	Brisbane (count(%))	Total	Barcelona (count(%))	Brisbane (count(%))	Total
**Lesion presence (per image)**						
With Lesions	2,412 (35.9)	4,311 (64.1)	**6,723**	3,633 (53.8)	3,117 (46.2)	**6,750**
Without Lesions	770 (44.0)	980 (56.0)	**1,750**	914 (52.8)	817 (47.2)	**1,731**
**Anatomic location (per image)**						
Torso	1,255 (39.0)	1,966 (61.0)	**3,221**	1,815 (56.3)	1,410 (43.7)	**3,225**
Left arm	486 (34.1)	940 (65.9)	**1,426**	731 (49.7)	740 (50.3)	**1,471**
Right arm	546 (37.1)	927 (62.9)	**1,473**	840 (59.1)	582 (40.9)	**1,422**
Left leg	460 (38.7)	730 (61.3)	**1,190**	571 (48.9)	597 (51.1)	**1,168**
Right leg	435 (38.0)	710 (62.0)	**1,145**	589 (51.1)	563 (48.9)	**1,152**
Unknown	0 (0.0)	18 (100.0)	**18**	1 (2.3)	42 (97.7)	**43**
**Sex at birth**						
Male	9 (39.1)	14 (60.9)	**23**	14 (58.3)	10 (41.7)	**24**
Female	13 (56.5)	10 (43.5)	**23**	11 (45.8)	13 (54.2)	**24**
Unknown	2 (66.7)	1 (33.3)	**3**	2 (66.7)	1 (33.3)	**3**
**Patient age groups**						
<30	0 (0.0)	0 (0.0)	**0**	2 (100.0)	0 (0.0)	**2**
30-39	4 (66.7)	2 (33.3)	**6**	2 (50.0)	2 (50.0)	**4**
40-49	5 (55.6)	4 (44.4)	**9**	6 (60.0)	4 (40.0)	**10**
50-59	8 (53.3)	7 (46.7)	**15**	9 (60.0)	6 (40.0)	**15**
60-69	1 (10.0)	9 (90.0)	**10**	1 (12.5)	7 (87.5)	**8**
≥70	4 (66.7)	2 (33.3)	**6**	5 (55.6)	4 (44.4)	**9**
Unknown	2 (66.7)	1 (33.3)	**3**	2 (66.7)	1 (33.3)	**3**
Total images	**8,473**			**8,481**		
Total annotations	**29,403**			**30,594**		

As Table 3 shows, the dataset maintains the same proportional distribution between Barcelona and Brisbane. The training set exhibits an approximate 4:1 ratio of lesion to non-lesion cases, comprising 6,723 lesion cases and 1,750 non-lesion cases. Similarly, the test set maintains a consistent ratio of approximately 4:1, with 6,750 lesion cases and 1,731 non-lesion cases. The anatomical distribution of the dataset reveals the torso as the primary examination site, accounting for approximately 38% of images in both the training and test sets. This is followed by a balanced representation of images depicting the arms and legs. Additionally, the sex distribution is notably balanced, with an almost equal proportion of male and female patients in both the training set (23 males and 23 females) and the test set (24 males and 24 females).

The age distribution reveals geographic variations, with cases under 30 being exclusively from Barcelona in the test set, while no cases under 30 exist in the training set. The 60-69 age group shows a strong presence in the Brisbane set (90% in the training and 87.5% in the test set). The middle age groups (40-59) show a relatively balanced distribution between the two locations. A small portion of cases (three patients in each set) have unknown age and sex information. The training set contains 49 unique patients with an average of 173 images per patient, while the test set includes 51 unique patients with an average of 166 images per patient. For images containing lesions, there is an average of four lesions per image in the training set and five lesions per image in the test set, as evidenced by the total annotation counts (29, 403 annotations across 6, 723 lesion images in the training set, and 30, 594 annotations across 6, 750 lesion images in the test set). All patients had white skin (Fitzpatrick skin type I-II) and were predominantly of European ancestry.

## Technical Validation

All annotated tiles were manually reviewed by a team of dermatologists to ensure the highest level of precision and reliability. These experts carefully evaluated the quality of the annotations carried out by Isahit’s *labellers* and *reviewers*, verifying their consistency and accuracy in representing the required details. In cases where discrepancies or inaccuracies were identified, the dermatologists corrected the annotations themselves to align with the expected standards. This rigorous quality control process was essential in maintaining the integrity of the dataset and ensuring that it met the requirements for subsequent analysis and research.

## Usage Notes

Pixel sizes varied across images due to the different angles and distances between the skin of the patient and the configuration settings of the VECTRA WB360 scanner at both data acquisition sites. To ensure accurate physical dimensions when analysing lesion sizes, users are advised to convert pixel measurements using the pixel spacing information provided in the metadata rather than relying on raw pixel counts. With the aim of protecting patient privacy, a name anonymisation process was implemented to ensure that multiple image tiles from the same patient remain untraceable. The dataset predominantly contains benign lesions, which users may observe when inspecting the types of lesions present in the images. While annotations underwent careful review, users should note that there may be occasional inconsistencies.

## Data Availability

To support research with this dataset, helper scripts have been provided in the iToBoS GitHub repository for tasks such as data loading, preprocessing, and annotation visualisation. Users seeking additional information should consult the repository documentation or contact the dataset maintainers.
